# Peri-implant diseases diagnosis, prognosis and dental implant monitoring: a narrative review of novel strategies and clinical impact

**DOI:** 10.1186/s12903-023-02896-1

**Published:** 2023-03-30

**Authors:** Rita Bornes, Javier Montero, André Correia, Tiago Marques, Nuno Rosa

**Affiliations:** 1 Faculty of Dental Medicine (FMD), Center for Interdisciplinary Research in Health (CIIS), Universidade Católica Portuguesa, Viseu, Portugal; 2 Department of Surgery, Faculty of Medicine, University of Salamanca, Salamanca, Spain

**Keywords:** Peri-implant diseases, Biomarkers, Molecular diagnosis, Prognosis, Precision dental medicine, Point-of-care test

## Abstract

**Background:**

The diagnosis of peri-implantar and periodontal relies mainly on a set of clinical measures and the evaluation of radiographic images. However, these clinical settings alone are not sufficient to determine, much less predict, periimplant bone loss or future implant failure. Early diagnosis of periimplant diseases and its rate of progress may be possible through biomarkers assessment. Once identified, biomarkers of peri-implant and periodontal tissue destruction may alert the clinicians before clinical signs show up. Therefore, it is important to consider developing chair-side diagnostic tests with specificity for a particular biomarker, indicating the current activity of the disease.

**Methods:**

A search strategy was created at Pubmed and Web of Science to answer the question: “How the molecular point-of-care tests currently available can help in the early detection of peri-implant diseases and throws light on improvements in point of care diagnostics devices?”

**Results:**

The PerioSafe® PRO DRS (dentognostics GmbH, Jena) and ImplantSafe® DR (dentognostics GmbH, Jena ORALyzer® test kits, already used clinically, can be a helpful adjunct tool in enhancing the diagnosis and prognosis of periodontal/peri-implantar diseases. With the advances of sensor technology, the biosensors can perform daily monitoring of dental implants or periodontal diseases, making contributions to personal healthcare and improve the current status quo of health management and human health.

**Conclusions:**

Based on the findings, more emphasis is given to the role of biomarkers in diagnosing and monitoring periodontal and peri-implant diseases. By combining these strategies with traditional protocols, professionals could increase the accuracy of early detection of peri-implant and periodontal diseases, predicting disease progression, and monitoring of treatment outcomes.

**Supplementary Information:**

The online version contains supplementary material available at 10.1186/s12903-023-02896-1.

## Background

Periodontitis is an inflammatory oral disease clinically characterized by pathological deepening of the gingival sulcus, loss of attachment, and formation of periodontal pockets with supportive alveolar bone resorption [1]. The beginning and development of this disease is a result of an interaction between pathogenic bacteria in the subgingival dental biofilme and the host response. In general, periodontal tissue damage is gradual, characterized by periods of active and remission disease without clearly alarming symptoms. In cases of neglect, permanent periodontal damage may occur. Although, it is well established that periodontal and periimplantar inflammation is associated with the presence of certain bacteria [2]. Additional factors and clinical confounders have been identified specially smoking, previous periodontal disease, poor oral hygiene, and residual excess cement have all been associated with peri-implant diseases [3]. Recent studies have also focused on the prosthetic features such restoration emergence profile and angle, showing that over-contoured restorations have higher risk of developing periimplantitis [4].

Early diagnosis of gingivitis or mucositis is an effective way to reduce the risk of developing periodontitis or peri-implantitis, respectively [5, 6]. The diagnosis of peri-implantar and periodontal diseases is mainly based on an array of clinical measurements and pocket probing depths, bleeding on probing and assessment of radiographic images. These clinical parameters alone are not enough to determine active peri-implant disease, future crestal bone loss, or future implant failure. Additional information based on medical records is also essential, but it does not provide information to the current state of disease activity, nor do identify the individuals who are susceptible to future disease progression [7–9]. These conventional diagnostic protocols require several manual recordings, professional examiners with trained expertise, and the clinical data refers only to established disease states, not being able to predict before clinical signs set in [10].

Recently, a consensus from the European Federation of Periodontology (EFP) and the American Academy of Periodontology (AAP) proposed a new classification of periodontal diseases that consider the disease severity, extent and progression by applying a staging and grading system [11]. One of the goals of this new classification is to develop methods for accurate diagnosis and predicting the prognosis of peri-implant disease [12]. Therefore, this new classification scheme was designed to allow incorporation of changes in line with future developments such as diagnosis based on biomarkers.

Early diagnosis of peri-implant diseases and their rate of progression may be possible with the assessment of biomarkers. Once identified, biomarkers of peri-implant and periodontal tissue destruction may alert the clinicians before clinical signs set in. Combining those strategies with traditional protocols, professionals could increase the accuracy of early detection of peri-implant and periodontal diseases, the prediction of disease progression and monitoring of treatment effects [13–15]. Therefore, it is important to consider the development of diagnostic chairside tests with specificity for a particular biomarker, indicating the current activity of the disease.

The aim of this review is to identify and analyze how the molecular point-of-care (PoC) tests currently available can help in the early detection of peri-implant diseases and throws light on improvements in point of care diagnostics devices, such as lab-on-a-chip and biosensors.

## Methods

The methodology included applying a search strategy, defining inclusion and exclusion criteria, and retrieving studies; selecting studies; extract relevant data; and performing tables to summarize the results. Searches of PubMed and Web of Science were performed to gather literature published until September 2022. The search terms used follow in the Tables 1 and 2, and 3 (Supplement 1) according to the database used.

The inclusion criteria for selection were articles written in English, studies using saliva or crevicular fluid, studies which apply Omics sciences or artificial intelligence or novel approaches as predictive tool to ensure gingival, periodontal, or implant success. Exclusion criteria included any articles that failed to involve items described in the inclusion criteria or any article that described in vitro studies, studies using animals or nonoral tissues, studies using tools to predict non-implant treatment success, comparative microbiological technique studies, comparative and experimental studies with different materials design of dental implant surface or rehabilitation components.

The search strategy for this review involved 3 stages: reviewing titles, abstracts, and final selection of articles for full text analysis. Articles selected from the database search were sorted independently by 2 reviewers (R.B. and N.R.), and any differences in selection were discussed with a third reviewer (A.C.). Upon the reviewers’ agreement, articles that did not meet the predetermined inclusion criteria were excluded. Abstracts of the articles selected at the second stage were independently evaluated by the same reviewers, and articles selected for final analysis were obtained in full text. At the final stage, the full text of the obtained articles was analyzed.

## The need of precision diagnostic, prognostic and monitoring indicators

It is consensual in the scientific community that implant success cannot be only evaluated based on implant survival and should also consider peri-implant conditions and crestal bone-level stability. It is generally accepted that initial remodeling of peri-implant bones occurs due to the biological adaptation of peri-implant tissues, and subsequent tissue stabilization is expected [12]. The clinicians keep it as a normal bone remodeling process, however, an unstable bone can cause different problems, leaving the clinician uncertain, if the implant will be stable for longer. For this reason, clinician’s duty is to seek as least bone loss as possible [16].

Peri-implantitis is an inflammatory disease that occurs in tissues around dental implants, characterized by progressive loss of supporting bone. According to the Consensus report of workgroup 4 of the “2017 World Workshop on the Classification of Periodontal and Peri‐Implant Diseases and Conditions”, peri‐implant health is characterized by the nonexistence of erythema, bleeding on probing, swelling, or suppuration. It is not possible to define a range of probing depths compatible with health. Peri‐implant health can exist around dental implants with reduced bone support [2].

All the scientific evidence, as well as the clinical assessment used nowadays by clinicians is strictly based on clinical, analytical, and radiographic parameters which, indeed, provide limited information to deal with the multi-factorial complexity of implant-supported rehabilitation procedures. Furthermore, from the point of view of diagnosing and staging peri-implant diseases, those methods can only register the pre-existent state and not the current condition itself, not considering the patient’s clinical condition. Moreover, it does not contemplate systemic conditions, lifestyle, hormonal changes, and ageing, among other aspects, related to individual inflammatory processes which may consequently influence the local immunological response. In other hand, for any clinician, the greatest challenge is predicting the success of rehabilitation or the identification of patients with high risk of disease [17].

In this way, there is the necessity to create diagnoses supported by precise and standardized approaches such as omics sciences. Omics technologies have emerged as a powerful tool to investigate different molecular mechanisms between health and disease states. Molecules such as biomarkers are often used in medicine to accurately determine the state of the disease or responses to a treatment and contribute to find the targets of new therapies [11, 17]. This strategy is increasingly being considered in the literature as a future protocol to be implemented in monitoring of peri-implant disease.

Thus, the peri-implant treatment would not only be an intensive local treatment and transversal to all individuals, but a more individualized treatment. This suggests a more embracing treatment, such as usual local debridement and disinfection protocols, but also give relevance to currently available systemically administered host modulation therapies. This type of protocol suggests that all patients rehabilitated with dental implants should be analyzed for well-established biomarkers systemic inflammation (for example high sensitivity C-reactive protein (hsCRP), cytokines such as interleukin-6 (IL-6), and collagenolytic enzymes such as MMP-8, MMP-9) in their biofluid samples, before and after local debridement procedures [18].

## Molecular markers

Currently, we are in the “emerging era of high-integrated precision diagnostics” [19]. Although blood remains the most used biofluid sample, saliva has the potential to achieve a more relevant role on the diagnosis of pathologies. It has many advantages: it’s an easy and fast collection method, and a non-invasive technique to collect the sample. As so, it may play a major role as a diagnostic biofluid especially in children and non-cooperative people [20]. Saliva has been proposed as a diagnostic fluid not only for oral diseases, such as caries, periodontitis [21, 22] and oral cancer, but also for systemic diseases, including diabetes [23], autoimmune, viral [24] bacterial and cardio- vascular diseases [15].

Literature presents several research to identify biomarkers associated with peri-implant disease. Up to the present date, different molecules have been investigated because of their molecular roles in inflammation or in tissues damage [25, 26]. Since there are numerous molecules identified in the literature related to biological mechanisms of peri-implantitis, in this study we only include the most actual, and already in use, biomarkers for peri-implantitis diagnostic point-of-care tests.

A meta-analyses that combined seven researches determined that interleukin-1beta (IL-1β) and tumor necrosis factor‐alpha (TNF-α), can be used as supplementary criteria for diagnosis of peri-implant infection, although cannot be used to distinguish peri-implant mucositis from peri-implantitis[27]. Ramseier et al. [28] reported biomarker assessment at teeth and implants in hundreds of patients 10 years after implant placement. Concerning IL-1β, it was observed significant differences between periodontal and peri‐implant conditions. Indeed, IL-1β was elevated in peri‐implantitis tissues and associated with increased probing depths. In the same study, the matrix metalloproteinase‐8 (MMP-8) demonstrated a trend similar to IL-1β, elevated in peri-implantitis and correlated with clinical parameters, such as bleeding on probing and increased probing depth [28].

Recently, Xanthopoulou et al. [29] revealed statistically significant differences of active matrix metalloproteinase-8 (aMMP-8) levels between healthy groups and the mucositis and peri-implantitis groups, and between the mucositis and the peri-implantitis groups. They demonstrated that elevated probing depths and aMMP-8 levels were significantly correlated. This information suggests that the aMMP-8 PoC test can be a helpful tool for early identification and screening of the risk of peri-implant diseases and progression. Also, Hentenaar et al. [30] compared biomarker levels in peri-implant crevicular fluid (PICF) of healthy implants with levels in PICF of implants with peri-implantitis. Levels of IL-1β and MMP-8 were significantly elevated in implants with peri-implantitis. No difference in levels of TNF-α, interleukin-6 (IL-6), monocyte chemoattractant protein-1 (MCP-1) and macrophage inflammatory protein-1α (MIP-1α), osteoprotegerina (OPG) and granulocyte colony-stimulating factor (G-CSF) between healthy and diseased implants was found. They also concluded that implants with peri-implantitis have higher levels of interleukin − 1β (IL-1β) and aMMP-8 in PICF compared to healthy implants.

Connective-tissue degradation and loss of attachment in periodontitis and peri‐implantitis diseases is due to matrix metalloproteinases. Among different matrix metalloproteinases and tissue inhibitor of metalloproteases, the aMMP‐8 has been selected as a more promising diagnostic tool [31].

## Molecular tests on the market for peri-implantitis

Routine monitoring of dental implants is nowadays crucial to prevent biological complications or failures. There are guidelines and consensus that validate and standardize clinical and radiographic assessment methods for the diagnosis of peri-implant diseases [2]. Although there are no standard protocols for diagnostic molecular tests, several scientific studies suggest that such tests might be useful to identify risk factors associated with developing peri-implant diseases, thus favoring early diagnosis [15]. It is expected that these tests have a high specificity and sensitivity which could be used chairside in a dental clinic or in a home use device [13].

There is a consensus in the literature that it is necessary to implement molecular diagnostic tests using biomarkers to identify early peri-implant disease. It is considered in the literature that MMP-8 is a biomarker of significance in the new classifications of periodontitis and peri-implantitis [9, 18, 32–36]. More notably, in oral fluids MMP-8 can also serve as a predictive and preventive adjunctive biotechnological tool, avoiding or reducing the evolution of gingivitis or mucositis to periodontitis or peri-implantitis, respectively [32, 37, 38].

Recently, two PoC chairside test kits have been developed - PerioSafe® PRO DRS (dentognostics GmbH, Jena) and ImplantSafe® DR (dentognostics GmbH, Jena) - to identify the presence of active MMP-8 on the saliva samples. The kits are like a COVID test or a pregnancy test, providing two lines of results indicating a higher risk of periodontitis/peri-implantitis. The advantages of these tests are that they are inexpensive, noninvasive, do not require specialized equipment or trained staff and provide a quick result with high sensitivity and specificity [39].

Once the presence of active MMP-8 has been identified in the sample, a quantitative analysis can be performed using the PerioSafe® PRO DRS (dentognostics GmbH, Jena) and ImplantSafe® DR (dentognostics GmbH, Jena) ORALyser, which is already a commercially available quantitative reader-based on aMMP-8 oral fluid specific point-of-care/chair-side lateralflow reader-equipped immunotests[40]. The results can be both qualitative and quantitative use the ORALyser reader [9, 34, 35, 40–42].

These tests have been validated in Finland, Nigeria, Germany, Holland, Malawi, Turkey, Sweden, and USA [31, 32, 43, 44]. The tests have diagnostic sensitivity and specificity 76–90% and 96%, respectively, corresponding to odds ratio of > 72 [31, 32]. The test results are quantitatively available by the reader in 5 min PoC/chair-side. The tests have been shown to be useful to screen susceptible sites and patients, differentiate active and inactive periodontitis and peri-implantitis sites, predict the future disease progression, and monitor the treatment.

The PerioSafe® PRO DRS (dentognostics GmbH, Jena) and ImplantSafe® DR (dentognostics GmbH, Jena) aMMP-8-POCT kits are efficient tools in improving the accuracy of diagnostic and prognostic of periodontal or peri-implant diseases and they are commercially available and approved technologies by the FDA on the United States of America and European Union [13, 41, 45]. PerioSafe® PRO DRS (dentognostics GmbH, Jena) and ImplantSafe® DR (dentognostics GmbH, Jena) and ORALyzer® tests have already been validated to function with a single biomarker, such as, aMMP-8 that is demonstrated as a biomarker of significance in the new classifications of both periodontitis and peri-implantitis. It is available as a mouthrinse (PerioSafe®PRO DRS (dentognostics GmbH, Jena)) and sulcular fluid/gingival crevicular fluid (ImplantSafe®DR (dentognostics GmbH, Jena)) variants [33, 35, 39–41]. The difference between both tests is that PerioSafe®PRO DRS (dentognostics GmbH, Jena) indicate the general periodontal status, whereas the ImplantSafe®DR(dentognostics GmbH, Jena) variant can be used as a site-specific test. Both have the advantage of being an easy-to-use tool-kit, and the possibility that either the patients themselves or general clinicians could interpret the result and understand whether or not they should refer the patient to a dentist [34].

## Advances in point-of-care devices

Future development of these PoC test kits should ideally consider the ASSURED criteria for the characteristics of PoC devices introduced by the World Health Organization (WHO). This requires that such devices should be “affordable, sensitive, specific, user friendly, rapid, and robust, with no complex equipment and deliverable to end-users” [13].

Recently, the progress in several informatic fields especially in biotechnologies allowed the development of biosensors and microelectro-mechanical systems (MEMS). Biosensors usually contains a “bioreceptor” unit responsible for selective recognition of the target and contains a physiochemical transducer able to translate the biorecognition into a signal that are sent to reader devices via electrical signals [46]. Those devices were developed for measurements in laboratories, or in a point-of-care (PoC) settings, or even for single-use home testing [47]. However, a new PoC technology - Lab-on-a-Chip (LOC) - has been developed integrating numerous laboratory assays in a single device, including process and preparation of the sample, identifications and quantification multiple biomarkers, and analysis [48].

Noninvasive biosensors have been developed to detect target analytes in several biological fluid, such as, saliva. Wearable saliva biosensors have progressed considerably in recent decades. They start to be incorporated into dentures and then into teeth. Their application has several targets, such as dental disease monitoring, biochemical monitoring in saliva, and food intake monitoring [49]. The detect data can be transmitted wirelessly to nearby device, and then interpreted by the individual or sent directly to the clinician to check in real-time the status of his patient [50, 51].

Although there are several fields in dentistry where biosensors can be applied as real-time diagnostic strategies, such as in the identification of caries and force exerted during orthodontic treatment, nonetheless it will be discussed in detail how biosensors can help in diagnosis and monitoring of dental implants [52, 53].

Hassanzadeh et al. [52] designed a capacitive sensor to evaluate the new bone growth around the dental implant. PEEK (Poly-ether-ether-ketone) was used for the creation of the sensor and its capacitance depended on the density and growth of bone around it. During the process of bone remodeling and osseointegration, the capacitance of the sensor would gradually be reduced to a seventh of the initial value. The capacitance data was then transmitted wirelessly to the external device and converted to the readable format for dentists. The merit of this sensor is that the capacitance of the sensor is chosen as a readily detectable indicator to manifest the condition of bone anchorage all the time with low energy consumption and wireless transmission. But as a disadvantage, the sensor cannot be removed after osteointegration, therefore the potentially harmful long-term effects of the sensor should be investigated deeply [52, 53].

It is consensual that the lifetime of dental implants can easily exceed 10 years, but there are many adverse influence factors related to its biological and mechanical problems [54]. As already defined in this review, the conventional diagnosis of peri-implant diseases is based on clinical signs, which are subjective, lack precision, and are time-delayed [53]. Diagnoses and treatments out of time, allow diseases such as peri-implantitis to develop, give rise to implant failure and needs of follow-up appointments, with invasive treatments, which will bring burden and pain to patients and waste medical resources [55].

To deal with the problem of timely notice and diagnosis of peri-implant diseases, Jeffrey et al. [53] reported a dental implantable temperature sensor for monitoring peri-implant diseases and increasing the lifetime of rehabilitations. Recognizing that temperature is one of the inflammation signs, it can be a relevant indicator to monitor the peri-implant tissues. Therefore, a multi-channel temperature sensor was created based on a photo-definable polyimide. The sensor was small and flexible to adhere to the abutment of dental implants. It was shown that this sensor had high stability, repeatability, linearity, and accuracy, and can send early warning signals when peri-implant diseases occur. However, for the aim of monitoring and alerting peri-implant diseases in more than 10 years, biological security, stability and the lifetime of the sensor should be improved to identify the needs of users [53].

Furthermore, implants and prosthetic structures are connected by connection screws that can be loosed sometimes and may lead to micro-displacement between implants and prosthetic structures eventually. Such micro-displacements may also result in the failure of dental implants. To increase the rate survival of dental implants, Sannino et al. [56] proposed a system to warn micro-displacements of the implant-prostheses connection. The system consisted of a micro-displacement sensor and wireless communications that can be put inside the prostheses. The micro-displacements data was wirelessly transmitted to the external unit. This sensor not only indicated the micro-displacements of dental implants, but also provided a platform to study the loading forces of dental implants and solve other problems [57]. But the adaptability and stability of this implantable system in the oral cavity should be further studied and improved.

Dental sensors were created to monitor the dental implants and extend their survival rate. Considering the dental sensor is often integrated on dental implants and must be kept in the body for years, the stability and safety of dental sensors need further investigations.

## Conclusion

Based on the findings, more and more emphasis is given on the role of biomarkers to recognize of present periodontal or peri-implantar status, as well as disease progression and response to therapy. The PerioSafe®PRO DRS (dentognostics GmbH, Jena) and ImplantSafe®DR (dentognostics GmbH, Jena) /ORALyzer® test kits, already used clinically, can be a helpful adjunct tool in enhancing the diagnosis and prognosis of periodontal or peri-implantar diseases.

In the foreseeable future, with the advances of sensor technology, the biosensors can perform daily monitoring of dental implants or periodontal diseases, making contributions to personal healthcare by the clinicians and moreover improve the current status quo of health management and human health.

## Electronic supplementary material

Below is the link to the electronic supplementary material.

Supplementary Tables

## Data Availability

Not applicable.

## References

[1] Mombelli A, Lang NP (1998). The diagnosis and treatment of peri-implantitis. Periodontol 2000.

[2] Lang NP, Berglundh T, Working Group 4 of Seventh European Workshop on Periodontology (2011). Periimplant diseases: where are we now? - Consensus of the seventh european workshop on Periodontology. J Clin Periodontol.

[3] Katafuchi M, Weinstein BF, Leroux BG, Chen Y-W, Daubert DM (2018). Restoration contour is a risk indicator for peri-implantitis: a cross-sectional radiographic analysis. J Clin Periodontol.

[4] Yi Y, Koo K, Schwarz F, Ben Amara H, Heo S (2020). Association of prosthetic features and peri-implantitis: a cross‐sectional study. J Clin Periodontol.

[5] Murakami S, Mealey BL, Mariotti A, Chapple ILC (2018). Dental plaque-induced gingival conditions. J Periodontol.

[6] Aral CA, Kesim S, Greenwell H, Kara M, Çetin A, Yakan B (2015). Alveolar bone protective and hypoglycemic Effects of systemic Propolis Treatment in Experimental Periodontitis and Diabetes Mellitus. J Med Food.

[7] Kinane DF, Stathopoulou PG, Papapanou PN (2017). Periodontal diseases. Nat Rev Dis Primer.

[8] Sahingur SE, Cohen RE. Analysis of host responses and risk for disease progression. Periodontol 2000. 2004; 34:57–83 10.1046/j.0906-6713.2002.003425.x 14717856

[9] Lahteenmaki H, Umeizudike KA, Heikkinen AM, Raisanen IT, Rathnayake N, Johannsen G (2020). aMMP-8 point-of-Care/Chairside oral Fluid Technology as a Rapid, Non-Invasive Tool for Periodontitis and Peri-Implantitis Screening in a Medical Care setting. Diagnostics.

[10] Hernndez M, Vernal R, Sorsa T, Tervahartiala T, Mntyl P, Gamonal J (2012). The role of Immuno-Inflammatory response in the Pathogenesis of Chronic Periodontitis and Development of Chair-Side Point of Care Diagnostics. Pathog Treat Periodontitis.

[11] Tonetti MS, Greenwell H, Kornman KS (2018). Staging and grading of periodontitis: Framework and proposal of a new classification and case definition. J Periodontol.

[12] Coli P, Christiaens V, Sennerby L, Bruyn HD. Reliability of periodontal diagnostic tools for monitoring peri-implant health and disease. Periodontol 2000. 2017; 73:203–217 10.1111/prd.12162 28000267

[13] Gul SS, Abdulkareem AA, Sha AM, Rawlinson A (2020). Diagnostic accuracy of oral fluids Biomarker Profile to determine the current and future status of Periodontal and Peri-Implant Diseases. Diagnostics.

[14] Alassy H, Parachuru P, Wolff L (2020). Peri-Implantitis diagnosis and prognosis using biomarkers in Peri-Implant Crevicular Fluid: a narrative review. Diagn Basel Switz.

[15] Carinci F, Romanos GE, Scapoli L. Molecular tools for preventing and improving diagnosis of peri-implant diseases. Periodontol 2000. 2019; 81:41–47 10.1111/prd.12281 31407432

[16] Puisys A, Auzbikaviciute V, Minkauskaite A, Simkunaite-Rizgeliene R, Razukevicius D, Linkevicius R (2019). Early crestal bone loss: is it really loss?. Clin Case Rep.

[17] Rakic M, Pejcic N, Perunovic N, Vojvodic D (2021). A Roadmap towards Precision Periodontics. Med-Lith.

[18] Golub LM, Räisänen IT, Sorsa T, Preshaw PM (2020). An unexplored Pharmacologic/Diagnostic strategy for Peri-Implantitis: a protocol proposal. Diagnostics.

[19] Davatzikos C, Rathore S, Bakas S, Pati S, Bergman M, Kalarot R (2018). Cancer imaging phenomics toolkit: quantitative imaging analytics for precision diagnostics and predictive modeling of clinical outcome. J Med Imaging.

[20] Kaufman E, Lamster IB (2002). The diagnostic applications of saliva - a review. Crit Rev Oral Biol Med.

[21] Rosa N, Correia MJ, Arrais JP, Costa N, Oliveira JL, Barros M (2014). The Landscape of protein biomarkers proposed for Periodontal Disease: markers with functional meaning. BioMed Res Int.

[22] Papale F, Santonocito S, Polizzi A, Giudice AL, Capodiferro S, Favia G (2022). The New Era of Salivaomics in Dentistry: frontiers and facts in the early diagnosis and Prevention of oral Diseases and Cancer. Metabolites.

[23] Soares A, Esteves E, Rosa N, Esteves AC, Lins A, Bastos-Filho CJA. An Analysis of Protein Patterns Present in the Saliva of Diabetic Patients Using Pairwise Relationship and Hierarchical Clustering. Springer-Verl. 202;148–159

[24] Esteves E, Mendes AK, Barros M, Figueiredo C, Andrade J, Capelo J (2022). Population wide testing pooling strategy for SARS-CoV-2 detection using saliva. PLoS ONE.

[25] Duarte PM, Serrao CR, Miranda TS, Zanatta LCS, Bastos MF, Faveri M (2016). Could cytokine levels in the peri-implant crevicular fluid be used to distinguish between healthy implants and implants with peri-implantitis? A systematic review. J Periodontal Res.

[26] Melguizo-Rodríguez L, Costela-Ruiz VJ, Manzano-Moreno FJ, Ruiz C, Illescas-Montes R (2020). Salivary biomarkers and their application in the diagnosis and monitoring of the most common oral pathologies. Int J Mol Sci.

[27] Faot F, Nascimento GG, Bielemann AM, Campão TD, Leite FR, Quirynen M. Can Peri-Implant Crevicular Fluid Assistin the Diagnosis of Peri-Implantitis? A Systematic Review and Meta-Analysis.J Periodontol. 2015;631–645 10.1902/jop.2015.140603 25675962

[28] Ramseier CA, Eick S, Brönnimann C, Buser D, Brägger U, Salvi GE. Host-derived biomarkers at teeth and implants in partially edentulous patients. A 10‐year retrospective study.Clin Oral Implants Res. 2016; 27:211‐217 10.1111/clr.12566 25682848

[29] Xanthopoulou V, Räisänen I, Sorsa T, Sakellari D. Active MMP-8 as a Biomarker of Peri-implant Health or Disease. 2022; 10.1055/s-0042-1753454. 10.1055/s-0042-1753454 PMC10569878 36063841

[30] Hentenaar DFM, De Waal YCM, Vissink A, Van Winkelhoff AJ, Meijer HJA, Liefers SC (2021). Biomarker levels in peri-implant crevicular fluid of healthy implants, untreated and non-surgically treated implants with peri-implantitis. J Clin Periodontol.

[31] Sorsa T, Sahni V, Buduneli N (2021). Active matrix metalloproteinase-8 (aMMP-8) point-of-care test (POCT) in the COVID-19 pandemic. Expert Rev Proteomics.

[32] Sorsa T, Gieselmann D, Arweiler NB, Hernández M. A quantitative point-of-care test for periodontal and dental peri-implant diseases.Nat Rev Primer2017; 3 10.1038/nrdp.2017.69 28905941

[33] Sorsa T, Bacigalupo J, Kononen M, Parnanen P, Raisanen IT (2020). Host-Modulation Therapy and Chair-Side Diagnostics in the treatment of Peri-Implantitis. Biosens-Basel.

[34] Räisänen IT, Sorsa T, Tervahartiala T, Raivisto T, Heikkinen AM (2021). Low association between bleeding on probing propensity and the salivary aMMP-8 levels in adolescents with gingivitis and stage I periodontitis. J Periodontal Res.

[35] Deng K, Pelekos G, Jin L, Tonetti MS (2021). Diagnostic accuracy of a point-of-care aMMP-8 test in the discrimination of periodontal health and disease. J Clin Periodontol.

[36] Öztürk V, Emingil G, Umeizudike K, Tervahartiala T, Gieselmann D-R, Maier K (2021). Evaluation of active matrix metalloproteinase-8 (aMMP-8) chair-side test as a diagnostic biomarker in the staging of periodontal diseases. Arch Oral Biol.

[37] Morais EF, Dantas AN, Pinheiro JC, Leite RB, Galvao Barboza CA, Vasconcelos Gurgel BC (2018). Matrix metalloproteinase-8 analysis in patients with periodontal disease with prediabetes or type 2 diabetes mellitus: a systematic review. Arch Oral Biol.

[38] Ziebolz D, Schmalz G, Gollasch D, Eickholz P, Rinke S (2017). Microbiological and aMMP-8 findings depending on peri-implant disease in patients undergoing supportive implant therapy. Diagn Microbiol Infect Dis.

[39] Al-Majid A, Alassiri S, Rathnayake N, Tervahartiala T, Gieselmann D-R, Sorsa T. Matrix Metalloproteinase-8 as an Inflammatory and Prevention Biomarker in Periodontal and Peri-Implant Diseases. Int J Dent. 2018; 2018:7891323 10.1155/2018/7891323 PMC6165625 30305812

[40] Alassiri S, Parnanen P, Rathnayake N et al. The Ability of Quantitative, Specific, and Sensitive Point-of-Care/Chair-Side Oral Fluid Immunotests for aMMP-8 to Detect Periodontal and Peri-Implant Diseases. Dis Markers. 2018; 2018:1306396 10.1155/2018/1306396 PMC6098860 30154936

[41] Sorsa T, Alassiri S, Grigoriadis A, Räisänen IT, Pärnänen P, Nwhator SO (2020). Active MMP-8 (aMMP-8) as a Grading and Staging Biomarker in the Periodontitis classification. Diagnostics.

[42] Sorsa T, Grigoriadis A, Sakellari D, Gupta S, Sahni V, Tervahartiala T (2021). On the accuracy, sensitivity, and grading of mouthrinse active matrix metalloproteinase-8 (aMMP-8) point-of-care testing (POCT). J Clin Periodontol.

[43] Johnson N, Ebersole JL, Kryscio RJ, Danaher RJ, Dawson D, Al-Sabbagh M (2016). Rapid assessment of salivary MMP-8 and periodontal disease using lateral flow immunoassay. Oral Dis.

[44] Lorenz K, Keller T, Noack B, Freitag A, Netuschil L, Hoffmann T (2017). Evaluation of a novel point-of-care test for active matrix metalloproteinase-8: agreement between qualitative and quantitative measurements and relation to periodontal inflammation. J Periodontal Res.

[45] Räisänen IT, Heikkinen AM, Pakbaznejad Esmaeili E, Tervahartiala T, Pajukanta R, Silbereisen A (2020). A point-of-care test of active matrix metalloproteinase-8 predicts triggering receptor expressed on myeloid cells-1 (TREM-1) levels in saliva. J Periodontol.

[46] Kim J, Campbell AS, de Ávila BE-F, Wang J (2019). Wearable biosensors for healthcare monitoring. Nat Biotechnol.

[47] Seker E, Sung JH, Shuler ML, Yarmush ML. Solving medical problems with BioMEMS. IEEE Pulse. 2011; 2:51 – 9. doi: 10.1109/MPUL.2011.942928. 10.1109/MPUL.2011.942928 PMC4490689 22147069

[48] Yılmaz B, Yılmaz F. Chapter 8—Lab-on-a-Chip Technology and Its Applications. In: Omics Technol. Bio-Eng. Academic Press. 2018; 145–153

[49] Arakawa T, Dao DV, Mitsubayashi K (2022). Biosensors and Chemical Sensors for Healthcare Monitoring: a review. IEEJ Trans Electr Electron Eng.

[50] Quadir NA, Albasha L, Taghadosi M, Qaddoumi N, Hatahet B (2018). Low-power implanted Sensor for Orthodontic Bond failure diagnosis and detection. IEEE Sens J.

[51] Lee Y, Howe C, Mishra S, Lee DS, Mahmood M, Piper M (2018). Wireless, intraoral hybrid electronics for real-time quantification of sodium intake toward hypertension management. Proc Natl Acad Sci.

[52] Hassanzadeh A, Moulavi A, Panahi A (2021). A New Capacitive Sensor for Histomorphometry evaluation of Dental Implants. IEEE Sens J.

[53] Kim JJ, Stafford GR, Beauchamp C, Kim SA (2020). Development of a Dental Implantable temperature sensor for real-time diagnosis of Infectious Disease. Sensors.

[54] Iacono VJ (2000). The Research, Science and Therapy Committee of the American Academy of Periodontology. Dental Implants in Periodontal Therapy. J Periodontol.

[55] Ramanauskaite A, Juodzbalys G (2016). Diagnostic Principles of Peri-Implantitis: a systematic review and guidelines for Peri-Implantitis diagnosis proposal. J Oral Maxillofac Res.

[56] Sannino G, Sbardella D, Cianca E, Ruggieri M, Coletta M, Prasad R (2016). Dental and Biological Aspects for the design of an Integrated Wireless warning system for Implant supported Prostheses: a possible Approach. Wirel Pers Commun.

[57] Flanagan D (2017). Bite force and dental implant treatment: a short review. Med Devices Evid Res.

