# Supplementary material for: Peri-implant diseases diagnosis, prognosis and dental implant monitoring: a narrative review of novel strategies and clinical impact
Source: BMC Oral Health. 2023 Mar 30;23:183. doi: 10.1186/s12903-023-02896-1 (PMC10061972; doi:10.1186/s12903-023-02896-1)
Supplement: Supplementary file 1 — Supplementary Tables [file 12903_2023_2896_MOESM1_ESM.docx]

**SUPPLEMENT 1**

Table 1. Research methodology on PubMed (MESH)

| **#1** | "Periimplantitis"[MeSH Terms] |
| --- | --- |
| **#2** | "Periodontitis"[MeSH Terms] |
| **#3** | "Saliva"[MeSH Terms] |
| **#4** | "Diagnostic test kit"[MeSH Terms] |
| **#5** | "Point of care"[MeSH Terms] |
| **#6** | “Chair-side diagnostic” [MeSH Terms] |
| **#7** | “Biosensor” [MeSH Terms] |
| **Research combination** | (#1 OR #2) AND (#3) AND (#4 OR #5 OR #7) |
| **Total number of articles** | **23 articles** |

Table 2. Research methodology on PubMed (natural language)

| **(periimplantitis OR periodontitis) AND (saliva) AND (diagnostic test kit OR point of care OR chair-side diagnostic OR biosensor)**  (("peri implantitis"[MeSH Terms] OR "peri implantitis"[All Fields] OR ("peri"[All Fields] AND "implantitis"[All Fields]) OR "peri implantitis"[All Fields] OR ("periodontal"[All Fields] OR "periodontally"[All Fields] OR "periodontically"[All Fields] OR "periodontics"[MeSH Terms] OR "periodontics"[All Fields] OR "periodontic"[All Fields] OR "periodontitis"[MeSH Terms] OR "periodontitis"[All Fields] OR "periodontitides"[All Fields])) AND ("saliva"[MeSH Terms] OR "saliva"[All Fields] OR "salivas"[All Fields] OR "saliva s"[All Fields]) AND ("reagent kits, diagnostic"[MeSH Terms] OR ("reagent"[All Fields] AND "kits"[All Fields] AND "diagnostic"[All Fields]) OR "diagnostic reagent kits"[All Fields] OR ("diagnostic"[All Fields] AND "test"[All Fields] AND "kit"[All Fields]) OR "diagnostic test kit"[All Fields] OR ("point of care systems"[MeSH Terms] OR ("point of care"[All Fields] AND "systems"[All Fields]) OR "point of care systems"[All Fields] OR ("point"[All Fields] AND "care"[All Fields]) OR "point of care"[All Fields]) OR ("chair-side"[All Fields] AND ("diagnosis"[MeSH Terms] OR "diagnosis"[All Fields] OR "diagnostic"[All Fields] OR "diagnostical"[All Fields] OR "diagnostically"[All Fields] OR "diagnostics"[All Fields])) OR ("biosensing techniques"[MeSH Terms] OR ("biosensing"[All Fields] AND "techniques"[All Fields]) OR "biosensing techniques"[All Fields] OR "biosensor"[All Fields] OR "biosensors"[All Fields] OR "biosensor s"[All Fields] OR "biosensoric"[All Fields] OR "biosensorics"[All Fields]))) | |
| --- | --- |
| **Total number of articles** | **113 articles** |

Table 3. Research methodology on Web of Science

| (Periimplantitis OR Periodontitis) AND (Saliva) AND (Diagnostic test kit OR Point of care OR Chair-side diagnostic OR Biosensor) | |
| --- | --- |
| **Total number of articles** | **79 articles** |
